# Genetic analysis for rs2280205 (A>G) and rs2276961 (T>C) in SLC2A9 polymorphism for the susceptibility of gout in Cameroonians: a pilot study

**DOI:** 10.1186/s13104-018-3333-6

**Published:** 2018-04-03

**Authors:** Jan René Nkeck, Madeleine Singwé Ngandeu, Vicky Ama Moor, Jériel Pascal Nkeck, Jean-Pierre Chedjou, Aude Laetitia Ndoadoumgue, Wilfred F. Mbacham

**Affiliations:** 1 Faculty of Medicine and Biomedical Sciences, The University of Yaoundé I, Yaoundé, Cameroon; 2 Rheumatology Unit of the Yaoundé Central Hospital, Yaoundé, Cameroon; 3 Biochemistry Laboratory of the Yaoundé University Hospital Centre, Yaoundé, Cameroon; 4 Laboratory of Public Health Biotechnology, Biotechnology Centre of the University of Yaoundé I, Yaoundé, Cameroon; 5 School of Health and Related Research, The University of Sheffield, Sheffield, UK

**Keywords:** Genetic, Gout, Non-synonymous variants, rs2280205, rs2276961, SLC2A9

## Abstract

**Objective:**

To determine the association of non-synonymous variants rs2280205 and rs2276961 of the SLC2A9 gene to gout in Cameroonians.

**Results:**

In a case–control study including 30 patients with acute gout matched to 30 healthy volunteers. We searched for polymorphism of the targeted variants using Restriction Fragment Length Polymorphism following polymerize chain reaction. Fisher exact test and Student t-test were used to compare variables, with a threshold of significance set at 0.05. The mean age of participants was 58 ± 8 years with 28 (93%) males. The family history of gout was found in one-third of the cases (p > 0.05). Uricemia was higher in cases than controls (p < 0.001) but 24 h urate excretion was similar in both groups (p > 0.05). Ancestral alleles (G and C) and their homozygous genotypes (GG and CC) of the targeted variants were predominant in both groups (p < 0.001). The polymorphisms of targeted variants were not associated with gout, and do not influence uric acid concentration in blood and urine. Non-synonymous variants rs2280205 and rs2276961 are not associated with gout in Cameroonians. However, the hereditary component of the disease suggests the influence of other genetic and/or environmental factors.

**Electronic supplementary material:**

The online version of this article (10.1186/s13104-018-3333-6) contains supplementary material, which is available to authorized users.

## Introduction

Gout is a major cause of arthritis worldwide especially for adult men [1]. Although it was previously considered rare in sub-Saharan Africa, recent studies show an increasing prevalence [1, 2]. In Cameroon, the global prevalence of gout is unknown, but hospital based studies showed an elevated frequency [2, 3]. Hyperuricemia which plays a central role in the pathogenesis of gout, mostly relies on genetic factors [4], and the SLC2A9 gene appears to be the most important of them [5–7]. Several studies have associated some non-synonymous variants of SLC2A9 gene to hyperuricemia and gout, especially rs3733591, rs6449213, rs16890979, rs1014290 and rs10489070 mostly in Caucasians, African-Americans and Asian populations [8–12]. Two non-synonymous variants, rs2280205 and rs2276961, which map the entry and exit of the GLUT9 canal isoform 2, a major uric acid transporter in the kidney, have been described as possible sites of interaction with urate and glucose thereby interfering with their excretion [13]. However, studies on theses variants do not give an association with hyperuricemia and gout [12, 14]. Studies in sub-Saharan Africa have revealed that patients with gout share the same clinical and paraclinical characteristics as those described the literature [2, 15]. However, data on the genetic predisposition of gout in this population remains unknown. The aim of this study was to determine whether the non-synonymous variants rs2280205 and rs2276961 of the SLC2A9 gene in Cameroonians have no influence on urinary excretion of uric acid and the risk of developing gout as seen in the Caucasian population.

## Main text

### Methods

#### Study design

It was a pilot study with a case–control design carried out from June to December 2017 in Yaoundé (Cameroon).

#### Study sample

Constituted of 30 patients with acute gout (cases) diagnosed according to the ACR criteria 1977 [16], and consulting at the Yaoundé Central Hospital. The controls were 30 healthy individuals, free of gout, and with normal uricemia. They were paired to each other, by age (± 2 years) and sex. Any participants known to be affected by at least one pathology that could modify uricemia such as: chronic kidney disease or any participants with a Glomerular Filtration Rate less than 60 mL/min/1.73 m^2^, treated or not cancer, treated or not chronic inflammatory disease, etc., were not included in this study. We did not include any person taking drugs which modified uricemia. The sample size was estimated using Cochran (1965:75) formula with 80% power and 5% error. The sampling was consecutive and non-exhaustive.

#### Data collection

For each participant, we collected clinical data including: age, sex, nationality, personal and family history of gout, eating habits; blood pressure, and anthropometric parameters. Hyperuricemic diet was defined as frequent consumption (at least twice in a week, during meals) of hyperuricemic foods. After clinical evaluation, we collected 5 mL of venous blood and 24 h urine. A drop of blood was placed on a sterile filter paper for each participant and stored in an envelope containing silica gel at 25 °C in a closed box. The secondary investigators who carried out the biological analysis on the samples were blinded.

#### Biochemical analysis

This involved assays for uricemia, 24 h of uraturia and serum creatinemia carried out at the Biochemistry Laboratory of the Yaoundé University Hospital Centre. Uric acid dosage was carried out by the Uricase enzymatic and colorimetric method [17]. Hyperuricemia was defined as uric acid levels above 60 mg/L for women and 70 mg/L for men [18]. The dosage of serum creatinine was made by the Jaffé kinetic method [17], and used to determine the Glomerular Filtration Rate using the MDRD (Modification of Diet in Renal Disease) formula with 4 parameters. Two technical replicates were performed for the biochemical analysis.

#### Molecular analysis

They were carried out at the Laboratory of Public Health Biotechnology of the Biotechnology Center of the University of Yaoundé I, using RFLP-PCR (restriction fragment length polymorphism-polymerase chain reaction). Primary DNA extraction on filter paper was performed by Chelex method; secondary DNA extracts were amplified with PCR using specific primers (Additional file 1: Table S1). Primers for each variant were designed using Oligo Calculator software (available at: http://www.pitt.edu/~rsup/OligoCalc.html). They were complementary to the DNA sequence, and their natural restriction site was identified using the New England Biolabs cutter software version 2.0 (available at: http://tools.neb.com/NEBcutter2/). Primers were designed by New England Biolabs. 22 µL of PCR mix for each gene (Additional file 2: Table S2) where added to 3 µL of DNA extract and submitted for 36 cycles on the PCR program (Additional file 3: Table S3) using a T3 Thermocycler designed by Biometra^®^. After amplification, amplicons were visualized after migration by electrophoresis on agarose gel (Additional file 4: Figure S1). The size of amplicons was estimated at 227 bp for rs2280205 and 334 bp for rs2276961, from the linear regression line of electrophoretic migration distances as a function of the natural logarithm of the size of the weight marker. Finally, 12 µL of amplicons were mixed with the restriction enzyme Msp1 designed by New England Biolabs, and specific to restriction sites for each variant (Additional file 1: Table S1). The digestion mix (Additional file 5: Table S4) was put on incubation at 37 °C overnight and then visualized after migration on electrophoresis with agarose gel (Additional file 6: Figure S2). The digested products were identified using electrophoretic migration distances (Additional file 1: Table S1). This permitted us to determine different alleles and genotypes. For rs2280205: minor and ancestral alleles were respectively A and G, and genotypes were homozygous AA and GG, and heterozygous AG; for rs2276961: minor and ancestral alleles were respectively T and C, and genotypes were, homozygous TT and CC, and heterozygous TC.

#### Statistical analysis

It was performed using S.P.S.S. software version 21.0. Quantitative variables were expressed in terms of mean and standard deviation. Qualitative variables were expressed in terms of counts and their proportions. The comparison of means of quantitative variables between the two groups was done by the Student’s t-test after groups tested for their normality. To compare means of more than 2 groups, we used the Kruskal–Wallis test. Qualitative variables were compared by Fisher’s exact test. The threshold of significance was set at 0.05.

### Results

#### Characteristics of the sample

Each group was made of 30 people of which 28 (93.3%) men. Participants were aged 58 ± 8 years for cases and 57.8 ± 8 years for controls. All affected women were menopausal. Family history of gout was found in one-third of patients with acute gout and this was similar for controls (p > 0.05) (Table 1). The Body Mass Index and blood pressure of participants of each group were quite similar (p > 0.05) (Table 1). Hyperuricemic diet and hyperuricemia were statistically associated to acute gout (p < 0.001). The excretion of uric acid in 24 h was similar for cases and controls (p > 0.05). Acute crisis on chronic gout was the clinical presentation in 22 (73.3%) cases.

**Table 1:** Baseline characteristics of sample

Variables	Cases (30)	Controls (30)	p value
Age (years)	58 ± 8	57.8 ± 8	> 0.05
Male	28 (93.3%)	28 (93.3%)	> 0.05
Family history of gout	9 (30%)	8 (26.6%)	> 0.05
Ascendant	5 (16.7%)	4 (13.3%)	
Descendant	4 (13.3%)	4 (13.3%)	
Hyperuricemic diet	24 (80%)	2 (6.7%)	< 0.001
Body mass index (kg/m^2^)	28.61 ± 4.5	27.25 ± 4.6	> 0.05
Systolic blood pressure (mmHg)	138 ± 17	132 ± 20	> 0.05
Diastolic blood pressure (mmHg)	85 ± 9	81 ± 10	> 0.05
Mean uricemia (mg/L)	81.2 ± 20.9	47.7 ± 11.8	< 0.001
Mean 24 h uraturia (mg/24 h)	544 ± 234	515 ± 229	> 0.05

#### Polymorphism of rs2280205 and rs2276961

Within the study sample, there was a marked predominance (p < 0.001) of ancestral alleles G and C for each variant (Table 2). Alleles A and T represented a small percentage. The most predominant genotypes in all groups were homozygous GG and CC (p < 0.001) (Table 2).

**Table 2:** Association of polymorphisms of study variants and gout

Polymorphisms	Cases (30)	Controls (30)	p value
rs2280205 alleles			
A	3 (10%)	1 (3.3%)	> 0.05
G	29 (96.7%)	30 (100%)	
rs2280205 genotypes			
AA	1 (3.3%)	0 (0%)	> 0.05
AG	2 (6.6%)	1 (3.3%)	
GG	27 (90%)	29 (96%)	
rs2276961 alleles			
T	10 (33.3%)	6 (20%)	> 0.05
C	26 (86.7%)	29 (96%)	
rs2276961 genotypes			
TT	3 (10%)	1 (3.3%)	> 0.05
TC	7 (23.3%)	5 (16.7%)	
CC	20 (66.7%)	24 (80%)	

#### Association of polymorphism and gout

Although the ancestral alleles and their homozygous genotypes were found to be predominant for both variants, they were not associated to gout (p > 0.05) (Table 2). The different alleles studied were not found to influence the levels of uric acid in blood and urine (p > 0.05) (Table 3).

**Table 3:** Influence of genotypes on uric acid levels in blood and urine

Genotypes	Uricemia (mg/L)	p value	24 h uraturia (mg/24 h)	p value
rs2280205				
AA	121.7	> 0.05	432	> 0.05
GG	63.4 ± 22		532 ± 233	
AG	73.8 ± 28.5		512 ± 252	
rs2276961				
TT	72.1 ± 37.8	> 0.05	512 ± 252	> 0.05
CC	64 ± 21.1		538 ± 240	
TC	65.7 ± 27.1		514 ± 195	

### Discussion

The prevalence of gout in sub-Saharan Africa is increasing with ageing of the population and the rise of comorbidities like type II diabetes, hypertension and chronic kidney disease. Determining the genetic risk factors for these diseases is a current challenge. In this preliminary study, we sought to determine the influence of two non-synonymous variants rs2280205 and rs2276961 of the SLC2A9 gene on the occurrence of gout in Cameroonians. It appears that the polymorphism of these variants did not influence the pathogenesis of gout.

Previous studies carried out on gout in Cameroon have set up its epidemiological and clinical characteristics. The present work is a pilot study using a targeted-gene approach to evaluate the effects of genetic predispositions on uric acid secretion. To the best of our knowledge, this is the first genetic study on gout in Cameroon. The selection criteria were stratified to select only cases of primary gout thereby limiting selection bias. Samples were collected and analyzed following guidelines which have been put in place for quality control. The observations of the basic characteristics of our sample are similar to those of patients affected by gout described in other studies in Cameroon, Africa and the world. Indeed, the patients in our study were people over 50 years old on average, with men significantly more affected. These values are close to those obtained by Singwé et al. as well as Kemta et al. in Cameroon [2, 3]. Hyperuricemia being the main factor in the development of gout explains why the uricemia of cases was statistically higher than controls. However, uric acid excretion in 24 h tend to be the same for cases and controls. As uric acid increase in blood, leading to gout, the normal renal response should be an increase in its urine excretion, as it is the main excreting pathway [19]. These results therefore suggest a possible defect in the renal excretion of uric acid in Cameroonians affected by gout.

The involvement of genetic factors in the pathogenesis of gout is supported by the inherited transmission of this condition [20, 21]. We found that one-third cases have at least one parent affected by gout. The GLUT9 transporter, is a channel that facilitates uric acid reabsorption from the proximal tubule with portions encoded by non-synonymous variants of SLC2A9 gene present on both ends [13]. These may constitute sites of potential interaction with the urate molecule that can facilitate its reabsorption and therefore lead to hyperuricemia; or with other SNP (Single nucleotide polymorphism), and then interfere in uric acid excretion [13, 22]. The role of these variants has not yet been studied in the African population. However, studies in Caucasians and African-Americans did not find an association with hyperuricemia and gout [12, 14]. We reported a large proportion of the ancestral alleles and their homozygous genotypes for the two targeted sequences in the study sample. These data differ from those of Li et al. and Chisnal, who found in shared proportion, the minor allele and the ancestral allele, and a predominance of the heterozygous genotype for the two targeted sequences [23, 24]. This discordance could certainly arise from ethnic differences in our population and suggests that a greater difference could be observed in the genetic repartition and genetic component of hyperuricemia and gout in this population compared to those described in the literature.

We found that these alleles were not associated to gout and that the uric acid levels in blood and urine were not modified by them. This could be as a result of the familial component found in similar proportions for both cases and controls which suggests a shared genetic inheritance and justifies the same distribution of ancestral and minor alleles and their homozygous genotypes given the same ethnic origin of both groups, which was not the case in previous studies [12, 23, 24]. However, although genetic factors play a major role in the pathogenesis and transmission of gout and determine the population at risk of developing gout, the role of the environment is equally important because it determines the individual risk of gout [21]. The disorders caused by these mutations on the urate transport could manifest later in life or in a context of hyperuricemic diet, as seen in cases but not controls. Thus, the follow up of people affected by these mutations in a cohort study will provide better answers concerning their implication.

This study reveals a large proportion of ancestral alleles and their homozygous genotypes of the rs2280205 and rs2276961 variants and suggests that they are not implicated in the pathogenesis of gout in Cameroonians. The existence of other genetic and environmental factors in this population is therefore suspected.

## Limitations

Limits to the interpretation of these results come from:

- The small sample size of the study, as a pilot study;
- The lack of strong criteria defining the hyperuricemic diet adapted in our context.

## Additional files

**Additional file 1: Table S1.** Primers for each variant, restriction enzyme and expected digested products.

**Additional file 2: Table S2.** Master mix for amplification of SLC2A9 variants.

**Additional file 3: Table S3.** Thermocycler program for amplification of SLC2A9 variants.

**Additional file 4: Figure S1.** Visualization of variants rs2280205 after amplification.

**Additional file 5: Table S4.** Master mix for digestion of SLC2A9 variants.

**Additional file 6: Figure S2.** Visualization of variants rs2280205 after digestion.

## Abbreviations

ACR: American College of Rheumatology
bp: base pairs
MWM: molecular weight marker
RFLP-PCR: restriction fragment length polymorphism-polymerize chain reaction
SLC2A9: solute carrier family 2 member 9
SNP: single nucleotide polymorphism
SPSS: statistical package for social sciences
