# Supplementary material for: Genetic analysis for rs2280205 (A>G) and rs2276961 (T>C) in SLC2A9 polymorphism for the susceptibility of gout in Cameroonians: a pilot study
Source: BMC Res Notes. 2018 Apr 3;11:230. doi: 10.1186/s13104-018-3333-6 (PMC5883404; doi:10.1186/s13104-018-3333-6)
Supplement: Supplementary file 1 — Additional file 1: Table S1. Primers for each variant, restriction enzyme and expected digested products. [file 13104_2018_3333_MOESM1_ESM.docx]

**Additional file 1: Table S1**: Primers for each variant, restriction enzyme and expected digested products.

| **Variants (mutation)** | **Primers** | **Restriction enzyme (site)** | **Digested product and genotypes** |
| --- | --- | --- | --- |
| **rs2280205 (**p.Pro321Leu) | Forward: CCTGTGCCTTAAAGGACCTTATG  Reverse:  CTCTCTGCTCATTCTTTCCTG | Msp1 (3’..C^CGG..5’) | AA: 227bp  AG: 227bp, 151bp, 76bp  GG: 171bp, 76bp |
| **rs2276961** (p.Gly25Arg) | Forward: GAGCATGCCAAGTCACACAGATGGA  Reverse: CACTGAGACCCATGGCAAGGAAACA | Msp1 (3’..CC^GG..5’) | TT: 334bp  TC: 334bp, 255bp, 79bp  CC: 255bp, 79bp |
