# Supplementary material for: Genetic analysis for rs2280205 (A>G) and rs2276961 (T>C) in SLC2A9 polymorphism for the susceptibility of gout in Cameroonians: a pilot study
Source: BMC Res Notes. 2018 Apr 3;11:230. doi: 10.1186/s13104-018-3333-6 (PMC5883404; doi:10.1186/s13104-018-3333-6)
Supplement: Supplementary file 2 — Additional file 2: Table S2. Master mix for amplification of SLC2A9 variants. [file 13104_2018_3333_MOESM2_ESM.docx]

Additional file 2: Table S2: Master mix for amplification of SLC2A9 variants.

| **Reagents** | | **rs2280205** | **rs2276961** |
| --- | --- | --- | --- |
|  |  | **Volumes (µL)** | **Volumes (µL)** |
| 1. **Nuclease free water** | | 16.75 | 18.25 |
| 1. **Thermopol buffer 10X** | | 2.5 | 2.5 |
| 1. **dNTP (10mM)** | | 0.5 | 0.5 |
| 1. **Primers (0,1µM)** | Forward | 1 | 0.25 |
|  | Reverse | 1 | 0.25 |
| 1. **Taq polymerase (1unit/µL)** | | 0.25 | 0.25 |
| **Total** | | 22 | 22 |
