# Supplementary material for: Genetic analysis for rs2280205 (A>G) and rs2276961 (T>C) in SLC2A9 polymorphism for the susceptibility of gout in Cameroonians: a pilot study
Source: BMC Res Notes. 2018 Apr 3;11:230. doi: 10.1186/s13104-018-3333-6 (PMC5883404; doi:10.1186/s13104-018-3333-6)
Supplement: Supplementary file 3 — Additional file 3: Table S3. Thermocycler program for amplification of SLC2A9 variants. [file 13104_2018_3333_MOESM3_ESM.docx]

Additional file 3: Table S3: Thermocycler program for amplification of SLC2A9 variants.

| **Steps** | **Temperature (°C)** | **Duration** |
| --- | --- | --- |
| 1. **Pre denaturation** | 94 | 5 minutes |
| 1. **Denaturation** | 94 | 30 seconds |
| 1. **Primers fixation** | 60 | 30 seconds |
| 1. **Elongation** | 72 | 30 seconds |
| **Repeat steps 2 to 4** | **35 times** | |
| 1. **Termination** | 72 | 7 minutes |
| 1. **Conservation** | 4 | 48h |
