# Supplementary material for: Genetic analysis for rs2280205 (A>G) and rs2276961 (T>C) in SLC2A9 polymorphism for the susceptibility of gout in Cameroonians: a pilot study
Source: BMC Res Notes. 2018 Apr 3;11:230. doi: 10.1186/s13104-018-3333-6 (PMC5883404; doi:10.1186/s13104-018-3333-6)
Supplement: Supplementary file 4 — Additional file 4: Figure S1. Visualization of variants rs2280205 after amplification. [file 13104_2018_3333_MOESM4_ESM.docx]

**
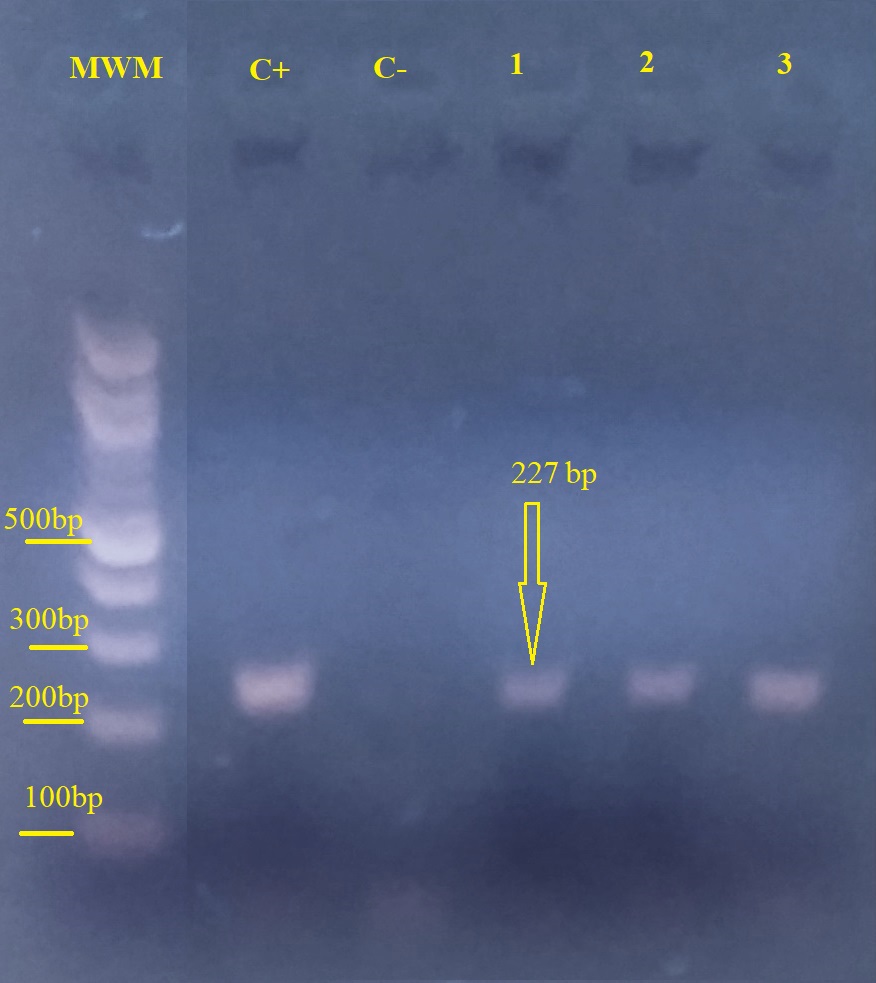
**

Additional file 4: Figure S1: Visualization of variants rs2280205 after amplification. *MWM: Molecular Weight Marker; C+: positive control; C-: negative control; 1,2,3: samples.*
