# Supplementary material for: Genetic analysis for rs2280205 (A>G) and rs2276961 (T>C) in SLC2A9 polymorphism for the susceptibility of gout in Cameroonians: a pilot study
Source: BMC Res Notes. 2018 Apr 3;11:230. doi: 10.1186/s13104-018-3333-6 (PMC5883404; doi:10.1186/s13104-018-3333-6)
Supplement: Supplementary file 5 — Additional file 5: Table S4. Master mix for digestion of SLC2A9 variants. [file 13104_2018_3333_MOESM5_ESM.docx]

Additional file 5: Table S4: Master mix for digestion of SLC2A9 variants.

| **Reagents** | **Volume (µL)** |
| --- | --- |
| 1. **Nuclease free water** | 8.6 |
| 1. **NEB cutter buffer 10x** | 3 |
| 1. **Restriction enzyme (Msp1)** | 0.4 |
| **Total** | **12** |
