# Supplementary material for: Genetic analysis for rs2280205 (A>G) and rs2276961 (T>C) in SLC2A9 polymorphism for the susceptibility of gout in Cameroonians: a pilot study
Source: BMC Res Notes. 2018 Apr 3;11:230. doi: 10.1186/s13104-018-3333-6 (PMC5883404; doi:10.1186/s13104-018-3333-6)
Supplement: Supplementary file 6 — Additional file 6: Figure S2. Visualization of variants rs2280205 after digestion. [file 13104_2018_3333_MOESM6_ESM.docx]

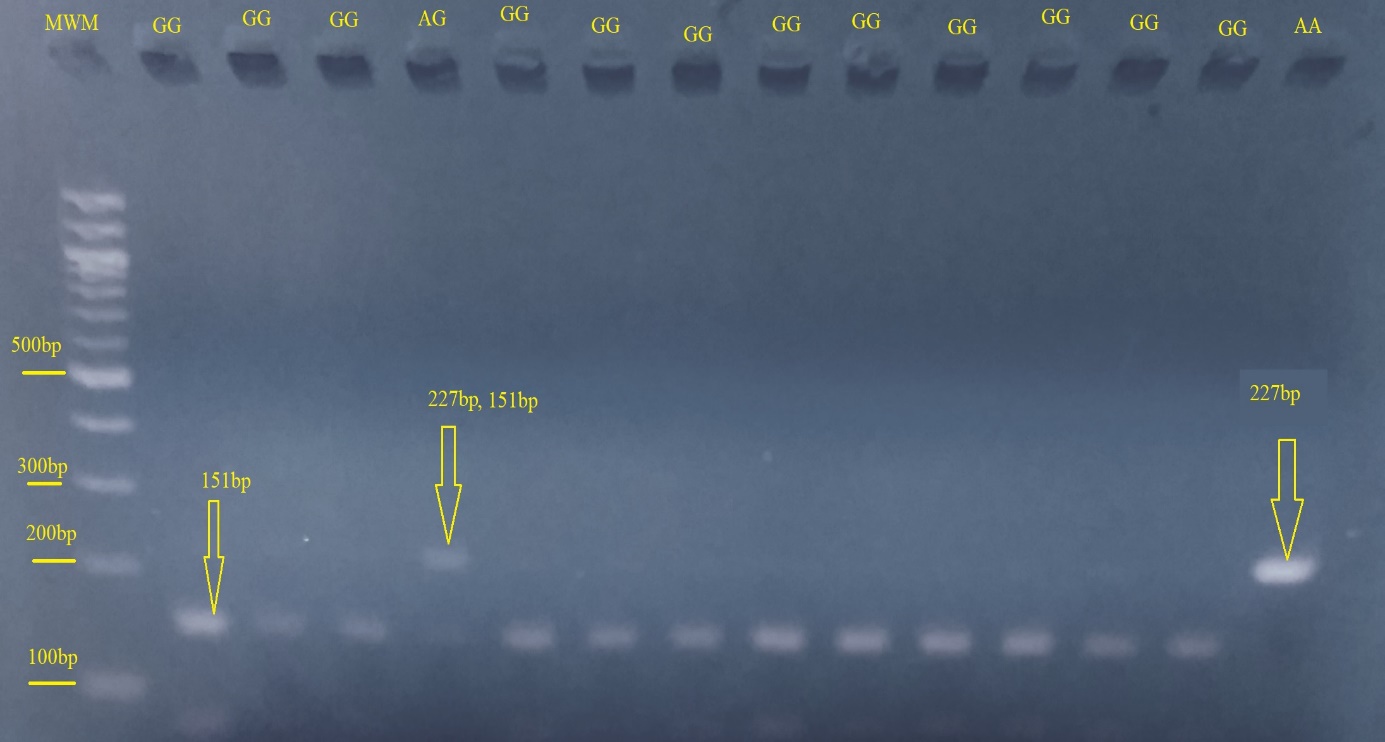


Additional file 6: Figure S2: Visualization of variants rs2280205 after digestion. *MWM: Molecular Weight Marker; GG, AG and AA represents the different genotypes.*
